# Supplementation with myo-inositol and Selenium improves the clinical conditions and biochemical features of women with or at risk for subclinical hypothyroidism

**DOI:** 10.3389/fendo.2022.1067029

**Published:** 2022-11-16

**Authors:** Juraj Payer, Peter Jackuliak, Martin Kužma, Matúš Džupon, Peter Vaňuga

**Affiliations:** ^1^ Comenius University Faculty of Medicine, 5th Department of Internal Medicine, University Hospital, Bratislava, Slovakia; ^2^ Medical Department, Exeltis, Bratislava, Slovakia; ^3^ National Institute of Endocrinology and Diabetology, Lubochna, Slovakia

**Keywords:** hypothyroidism, myo-inositol, Selenium, AITDs, TSH

## Abstract

**Purpose:**

The present study aims to evaluate the effect of myo-Inositol plus Selenium supplementation in patients affected by subclinical hypothyroidism.

**Methods:**

One hundred and forty-eight patients were included in the study from 8 different centers of Slovakia, and treated for 6 months with a daily dose of 600 mg myo-Ins plus 83 mcg Se. The patients included at the enrollment were women of reproductive age (18-50), who exhibit values of TSH in the range 2.5-5 mU/l and positivity to antibodies TPO-Ab/TG-Ab, or otherwise values of TSH in the range 5-10 mU/l both with and without positivity to antibodies TPO-Ab/TG-Ab.

**Results:**

Patients affected by subclinical hypothyroidism exhibited a significant improvement of their condition when treated for 6 months with a combination of myo-Inositol and Selenium. The TSH values significantly ameliorated along with the index of autoimmunity and the thyroid status. In a sub-class of patients, the auto-antibody titer decreased after myo-inositol + Selenium administration. The treatment also induces a regularization of the menstrual cycle and a reduction of the cholesterol in the patients enrolled for the study. Furthermore, a significant improvement is observed in the perception of the symptoms associated with subclinical hypothyroidism over the treatment period.

**Conclusion:**

A dietary supplementation with of myo-Inositol and Selenium in the treatment of patients affected by subclinical hypothyroidism exhibits a beneficial role in the recovery of TSH values, in the improvement of the symptoms associated to this condition and in the maintenance of the thyroid functions.

The trial was approved by the Ethical Committee from National Institute of Endocrinology and Diabetology of Lubochna, Slovakia, date 18.12.2018, registration number: 3124/2018.

## Introduction

The thyroid is a central actor of the endocrine system in the human body (1). Through the synthesis and secretion of thyroid hormones (TH) known as triiodothyronine (T3) and thyroxine (T4), it regulates several biological processes as neurological development, energy metabolism, cardiometabolic and reproductive system functions (2–5). Thyroid activity is regulated by the thyroid-stimulating hormone (TSH), which stimulates the production of the TH by the thyrocytes (6). The perturbation of the thyroid wellbeing and a diminished functioning could be a sign of early thyroid failure, which may lead to a pathological condition indicated as hypothyroidism (7). If untreated, it can evolve to serious health issues compromising the quality of life in the affected individuals (8).

Often, when the circulating level of TSH increases, even if the TH values remains normal, this condition may predict an early stage of hypothyroidism identified as sub-clinical hypothyroidism (SCH) (9). Condition of SCH might evolve to chronic pathology named overt hypothyroidism and, so far, the widest prescribed treatment to counteract those cases is Levothyroxine (LT4), a synthetic hormone administered to increase serum T4/T3 ratios and to normalize serum level of TSH (10, 11). The treatment of SCH, instead, is still debated, particularly if the patients affected exhibit a predisposition to other co-morbidities such as alterations of the menstrual cycle or metabolic alterations. The controversial recommendations regarding the treatment of SCH and the prescription of LT4 (12–14) is shifting the attention to alternative therapy as dietary supplements, that might efficiently counteract SCH conditions and as well avoid or post-pone the pharmacological treatment.

In the molecular network underpinning thyroid activity, myo-Inositol (myo-Ins) covers a pivotal role (1, 15). Particularly myo-Ins besides the recognized activity of insulin-sensitizer in diabetic scenarios (16, 17), increases the sensitivity of the thyrocytes to TSH, thus contributing to maintain thyroid physiology (18). Data retrieved from metabolic investigations described a higher demand of myo-Ins in subjects exhibiting hypothyroidism with respect to healthy individuals (19) and suggest that in hypothyroid patients the administration of exogenous myo-Ins might exert a positive activity on the thyroid functioning (20).

In some cases, a reaction of the immune system could lead to altered thyroid physiology, thus causing a persistent and chronic inflammatory state of the organ. This condition is classified as autoimmune thyroid disease (AITD) (21), which identify a clinical manifestation of lymphocytic infiltration of the thyroid gland, affecting thyroid function (22). Patients with AITD, often exhibit auto-antibodies such as anti-thyroglobulin (Tg-Ab) and anti-thyroid peroxidase (TPO-Ab) antibodies (23), and the same AITD is strongly associated with hypothyroidism condition (24–26). In this scenario, growing evidence indicates the use of myo-Ins as an effective approach to treat AITDs and SCH conditions, especially when its administration is associated with the Selenium (Se) (27–29). Indeed, Se is an essential micronutrient in the selenoprotein biosynthesis, and is crucial in metabolism, homeostasis, and regulation of thyroid hormones (30, 31). The presence of Se improves the efficacy of myo-Ins treatment in patients affected by AITDs, thanks to its recognized antioxidant and anti-inflammatory activity (32, 33). Even if the beneficial role of Se is well described and recognized in literature, the administration of Se in association with myo-Ins seems more effective in reducing the TSH levels in hypothyroid patients than the administration of the only Se (29, 34–36). The combined administration of myo-Ins plus Se seems effective to reduce the TSH and auto-antibody levels, thus enhancing thyroid wellbeing, and therefore restoring euthyroidism in patients diagnosed with AITD (37).

The absence of a reference treatment to counteract the features of SCH is prompting investigations on the potential effectiveness of a therapy based on micronutrients administration. In view of this, the aim of the present study is to evaluate whether the administration of myo-Ins associated with Se for the treatment of patients affected by SCH might be a beneficial approach to protect thyroid physiology by reducing the TSH values and the titer of the autoantibodies, and to improve the quality of life of the patients. As an association between thyroid dysfunction and menstrual cycle disturbances is not uncommon, particularly changes in menstrual cycle length and flux of bleeding are often highlighted in women with hypothyroidism (38), we included in the present study female individuals in order to evaluate the effect of the treatment with myo-Ins plus Se on the menstrual cycle regularity.

## Material and methods

Prospective interventional multicentric study. One hundred and forty-eight patients were initially included in the study from 8 different centers of Slovakia.

The inclusion criteria selected for the enrollment were as follows:

- Women of reproductive age (18–50);
- Values of TSH in the range 2.5-4.99 mU/l and positivity to antibody TPO-Ab/TG-Ab, or values of TSH in the range 5-10 mU/l with or without positivity to antibody TPO-Ab/TG-Ab.

The patients included in the study had TH in the reference range at baseline. The values of the TH were also measured at the end of the study and confirm to remain within the reference range.

By opposite the exclusion criteria were as follows:

- Serious co-morbidities (as diabetes or oncological diseases);
- Use of hormonal contraceptives;
- Pregnancy or women seeking pregnancy in the last 6 months before inclusion to survey;
- Treatment with thyroid hormones (LT4) in the last 6 months before inclusion to survey.

The patients were treated for 6 months with a daily dose of 600 mg myo-Ins plus 83 mcg Se. The values of the parameters evaluated during the study period, have been measured at baseline (T0), after 3 months of treatment (T3), and after 6 months of treatment (T6).

The patients enrolled in the study were given a questionnaire to investigate their perception of the symptoms associated to hypothyroidism over the treatment period. Particularly the symptoms investigated were as follows: level of fatigue, changes in weight, warm/cold tolerance, memory impairment, swelling, and pins and needles. Patients ranked from 1 to 10 the intensity of the perceived symptoms. Scores 1-3 indicate perception of low entity; scores 4-7 indicate perception of medium intensity; scores 8-10 indicate intense perception of the symptoms.

Additionally, the participants had the possibility to indicate in the questionnaire any side or undesired effects experienced following the treatment, and as well to report the days of missed therapy.

The primary outcome defined for the study was the changes in TSH value, while the secondary outcomes were: antibody titer (TPO-Ab, Tg-Ab), thyroid volume, AIT (autoimmune thyroiditis index), cholesterol levels, menstrual cycle regularity, and the quality of life of the patients.

Alterations in ultrasound images indicate the predisposition of a subject to develop an autoimmune thyroiditis. Score 1 is associated with changes in ultrasound images such as diffuse, moderately enlarged, hypoechoic gland with lobulated contours, heterogeneous echo pattern and fine, echogenic fibrotic streaks within. Score 0 indicates physiological ultrasound pictures Throughout the text these sonographic features are referred to as AIT index. AIT index with score= 1 means alteration of thyroid ultrasound, AIT index with score= 0 means no alteration of thyroid ultrasound. As indicator of risk factor for developing AITDs, we evaluated at each timepoint the percentage of patients exhibiting AIT score 1.

The overall volume of thyroid was obtained by summing up the values of the left and right lobe. The measures of the thyroid volume as well as the AIT assessment were carried out with an ultrasound evaluation.

Records of the baseline mean values of the parameters analyzed in the study are summarized in **Table 1**.

**Table 1:** Baseline Table.

Parameter	Value
Age of the population (years)	40 ± 9.6
Weight (kg)	69.9 ± 16.0
TSH level (μIU/ml)	4.4 ± 1.7
Thyroid volume (cm^3^)	11.7 ± 5.3
Cholesterol (mmol/L)	5.1 ± 2.4
Positive AIT (% of subjects)	62.2
Irregular mentrual cycle (% of subjects)	28.2

The tables reports the baseline values of the different parameters analyzed recorded at baseline.

A McNemar’s Test was adopted to evaluate the menstrual cycle regularity and the AIT changes. Paired T-test was performed to investigate thyroid volumes, TSH values, and cholesterol levels. The statistical analysis of the changes in the titer of the antibodies Tg-Ab and TPO-Ab was calculated with a Wilcoxon signed rank sum test and with a Chi Square Test.

The trial was approved by the Ethical Committee from National Institute of Endocrinology and Diabetology of Lubochna, Slovakia, date 18.12.2018, registration number: 3124/2018.

## Results

### TSH

The serum values of TSH were evaluated at T3 and T6. At T3, the patients exhibited a significant reduction in serum levels of TSH, with a median value changing from 4.7 μIU/ml to 4 μIU/ml (p<0.001). Analogously, at T6 the decrease observed was significant with respect to T0, with a median value of 3.8 μIU/ml (p<0.001). TSH values decreased also between T3 and T6, but without reaching significance.

The stratification of the TSH results was performed by fractioning the values in two groups:

- Group 1: TSH levels between 2.5 – 4.99 μIU/ml;
- Group 2: TSH levels between 5 – 10 μIU/ml.

**Figure 1:**
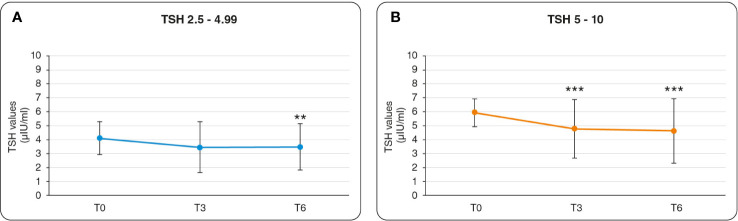
Graphics of changes in TSH values. **(A)** Results referred to Group 1: the variation of TSH in the range 2.5 – 4.99 (μIU/ml). Statistical relevance is assigned to the comparison of T3 versus T0, and of T6 versus T0. **(B)** Results referred to Group 2: the variation of TSH in the range 5 – 10 (μIU/ml). Statistical relevance is assigned to the comparison of T3 versus T0, and of T6 versus T0. ** = p value < 0.01, *** = p value <0.001.

In Group 1 reporting lower values of TSH, the treatment induced a significant reduction at T6 with respect to baseline from 4.1 – 3.4 μIU/ml (p<0.05) (**Figure 1**). The treatment induced a decrease in the levels of TSH already after 3 months compared to T0, but the variation was not significant.

In Group 2 reporting higher values of TSH, the treatment induced a significant reduction of the TSH values both at T3 and T6. The TSH values diminished respectively from 5.9 to 4.7 μIU/ml (p<0.05) at T3 vs T0, and from 5.9 to 4.6 μIU/ml (p<0.05) at T6 vs T0 (**Figure 1**). In both groups, the reduction observed in the TSH values from T3 to T6 resulted not significant.

### Overall thyroid volume

The measures of the thyroid volume at baseline with a median value of 10.3 cm^3^ increased at T3, reaching a median value of 11 cm^3^. At T6 the thyroid volume exhibits a recovery with a significant decrease compared to T3, reaching a median value of 10.3 cm^3^ (p<0.05).

### AIT changes

**Figure 2:**
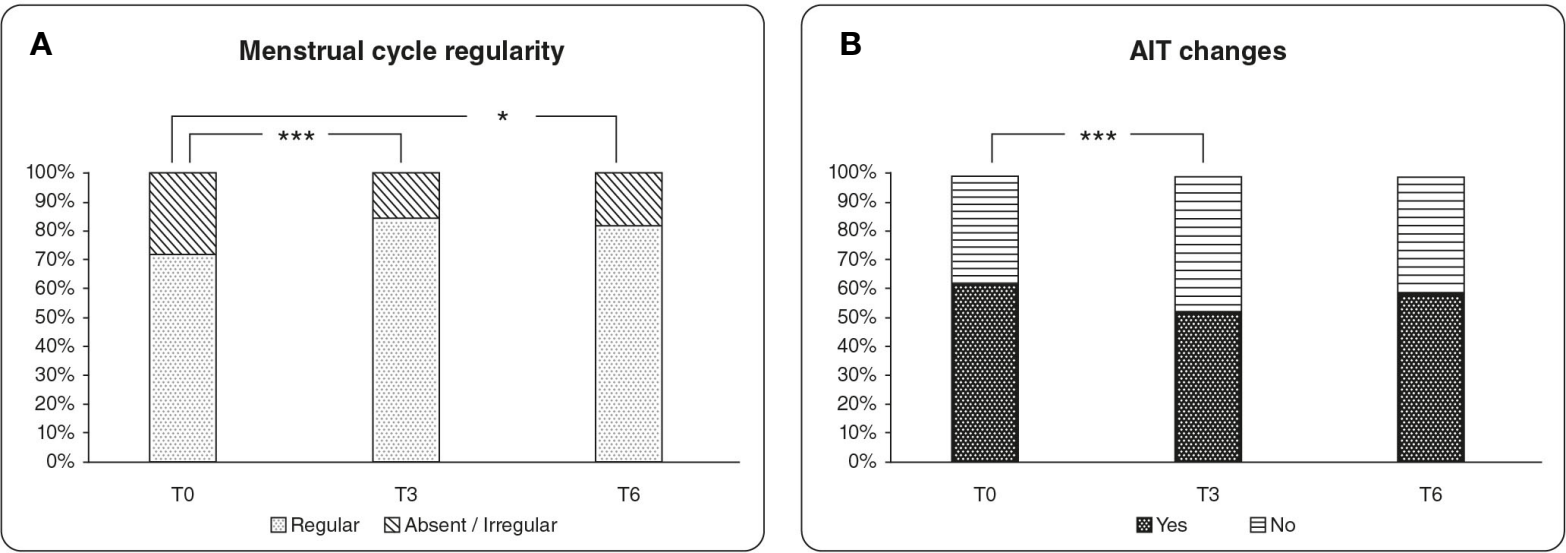
**(A)** Evaluation of menstrual cycle regularity over the treatment period. The graph reports the percentage values of patients with regular menstrual cycle compared with patients with absent or irregular menstrual cycle. **(B)** Evaluation of the AIT changes over the treatment period. The column chart represents the percentage values of patients with positive and negative AIT assessment. * = *p* value < 0.05, *** = *p* value <0.001.

At T3, the index of AIT changes improved since the proportion of patients with positive AIT changed from 62.2% to 51.9%, while proportion of patients with negative AIT increased from 37.8% to 48.2% (p<0.001). The same trend was observed at T6 (**Figure 2B**).

### TPO-Ab and Tg-Ab

The changes in the antibody titer were evaluated in those patients that had positivity for TPO-Ab and Tg-Ab at enrollment. In detail, 38 patients were positive to TPO-Ab (TPO-Ab > 34 IU/ml) and 48 patients were positive to Tg-Ab (Tg-Ab > 115 IU/ml). After 6 months of treatment, the titer of TPO-Ab significantly decreased at T6 in more than 60% of patients, with a median value of the variation of -21 IU/L (p<0.001). The treatment had no effect in the remainder of patients.

The treatment significantly improved also the Tg-Ab titer at T6 with respect to T0 in more than 57% of the patients, with a median value of the variation of -46 IU/L (p<0.001). The treatment had no effect in the remainder of patients.

### Cholesterol

Total cholesterol decreased already at T3 compared to T0, even if a significant reduction was observed only at T6, with median values changing from 4.9 mmol/L to 4.8 mmol/L (p<0.01).

### Irregular menstruation

A significant improvement of the menstrual cycle was observed after 3 months of treatment (p<0.001), with the percentage of women with regular cycle that increased from 71.8% to 84.6%, and the percentage of women with absent/irregular menstrual cycles that decreased from 28.2% to 15.4%. The improvement of the menstrual cycle remained significant also after 6 months of treatment (p<0.05) (**Figure 2A**), without significant differences between T3 and T6.

### Questionnaire

**Figure 3:**
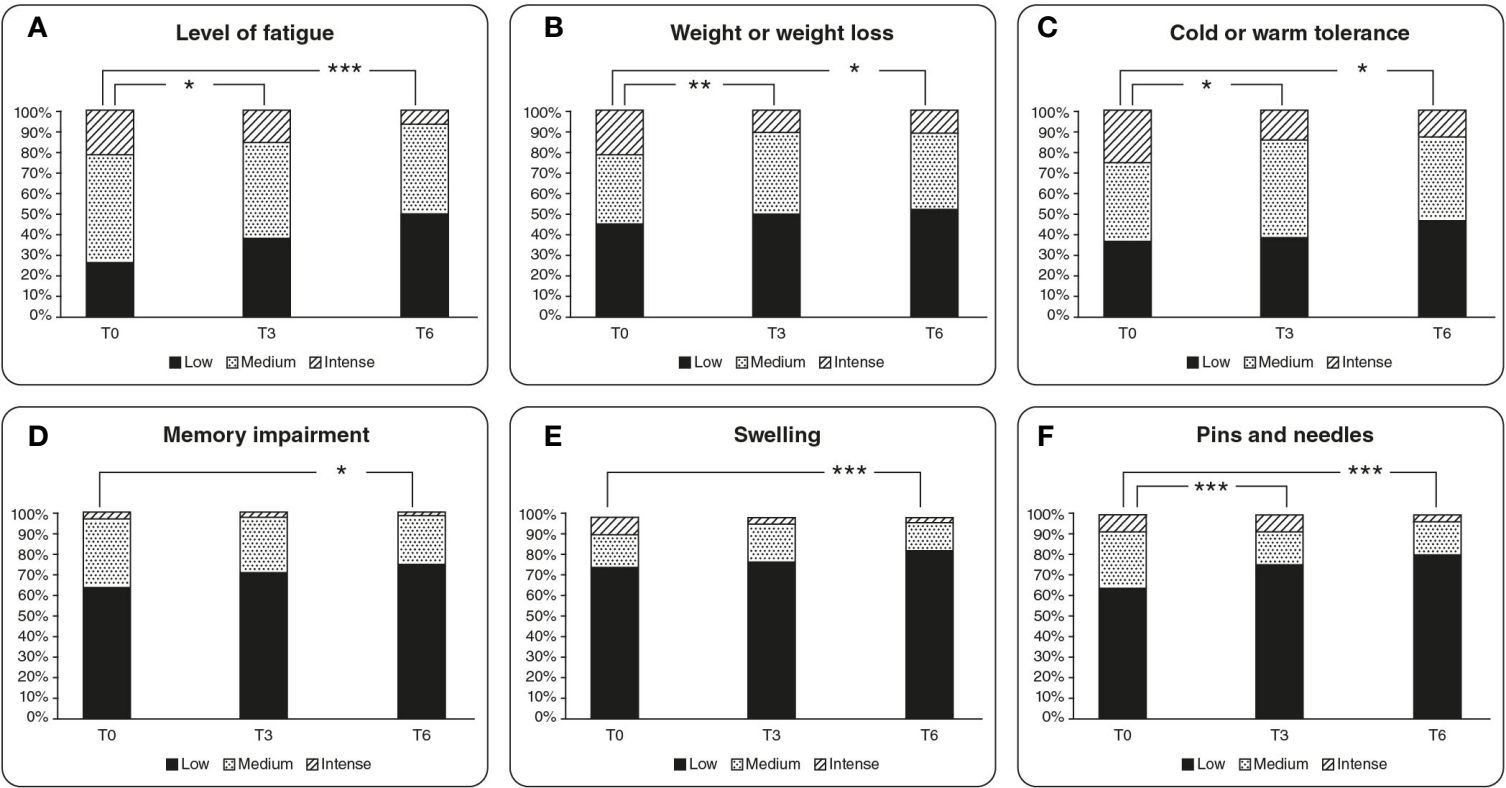
Graphical representation of results retrieved from the questionnaires regarding the quality of life of the patients. The graphs are referred to both 3 months and 6 months timepoints considered in the study, representing low, medium, and high perception of the sensation. **(A)** Level of fatigue trend over the treatment period, **(B)** changes in weight perception, **(C)** cold or warm tolerance, **(D)** memory impairment experienced, **(E)** swelling sensation over the 6 months of treatment, **(F)** sensation of pins and needles. * = *p* value < 0.05, ** = *p* value < 0.01, *** = *p* value <0.001.

- The level of fatigue significantly improved after 3 months. From T0 to T3, the patients ranking fatigue as 1-3 significantly increased from 27.1% to 39.9%, while those ranking the symptom as 8-10 significantly decreased from 21.1% to 15% (p<0.05). The result is confirmed at T6, with patients ranking 1-3 significantly increased reaching the 50.4% vs the 27.1% at baseline, while those ranking the level of fatigue as 8-10 significantly decreased from 20.9% to 6.9% (p<0.001) (**Figure 3A**).
- A significant improvement was observed for the perception of changes in weight at T3, with a percentage of patients ranking changes in weight of 1-3 significantly increased from 44.4% to 50.4%, while those ranking changes in weight as 8-10 decreased from 20.7% to 10.4% (p<0.01). At T6 the individuals ranking changes in weight as 1-3 significantly increased respect to T0 from 43.9% to 53.1%, while the percentage of patients scoring 8-10 exhibited a significant reduction from 20% to 10% (p<0.05) (**Figure 3B**).
- Warm/cold tolerance significantly improved during the treatment period. Indeed, at T3 vs T0 the percentage of patients ranking warm/cold tolerance as 1-3 increased from 37% to 38.5%, while the patients scoring warm/cold tolerance as 8-10 were significantly reduced from 25.2% to 14.8% (p<0.05). The same trend was observed at T6 vs T0, with a significant increase of participants ranking warm/cold tolerance as 1-3 from 37.2% to 46.5%, along with a significant decrease in the percentage individuals scoring warm/cold tolerance as 8-10 from 23.26% to 13.18% (p<0.05) (**Figure 3C**).
- At T3, no appreciable change in the perception of memory impairment was reported. However, it significantly improved at T6 compared to T0, with a percentage of patients ranking memory impairment as 1-3 increased from 65.1% to 76.7% and the patients ranking memory impairment as 8-10 were reduced from 3.1% to 1.6% (p<0.05) (**Figure 3D**).
- The evaluation of swelling perception resulted in a slight improvement at T3 even not statistically significant. Hence, the percentage of participants scoring swelling as 1-3 increased from 76.1% to 78.4% and the percentage of individuals ranking swelling as 8-10 was reduced from 8.2% to 3%. The improvement of the swelling perception became significant at T6 compared to T0, with a percentage of patients ranking swelling as 1-3 increased from 75.8% to 84.4%, while the percentage of individuals ranking swelling as 8-10 was reduced from 8.6% to 1.6% (p<0.01) (**Figure 3E**).
- The feeling of pins and needles significantly improved either at T3 and T6 compared to T0. In detail the percentage of patients ranking the feeling of pins and needles as 1-3 positively changed from 64.4% to 76.3% (p<0.01) while the percentage of participants ranking the feeling of pins and needles as 8-10 did not change at T3. Instead, at T6 the percentage of patients ranking the feeling of pins and needles as 1-3 significantly increased from 66.7% to 80.6%, while those ranking the perception of pins and needles as 8-10 significantly diminished from 7.8% to 3.1% (p<0.01) (**Figure 3F**).

## Discussion

In the present study we treated a group of patients with SCH with an association of myo-Ins and Se to evaluate their impact on the thyroid status, on the symptoms correlated with SCH and on the quality of life of the patients.

A deficit of thyroid functionality may lead to a pathological condition referred to as hypothyroidism. Mild or subclinical hypothyroidism is usually identified as a sign of early thyroid failure and features TSH values above the reference range (0.4 – 4 mIU/L) and TH levels within the normal range (39–42). In the present study we enrolled women exhibiting TSH values in the range 2.5-4.99 mU/l and positivity to antibody TPO-Ab/Tg-Ab, or women exhibiting values of TSH in the range 5-10 mU/l with or without positivity to antibody TPO-Ab/Tg-Ab. The selection of the study group was aimed to include patients with altered TSH values, which are identified as canonical SCH patients. Additionally, we also included patients with TSH in the reference range but exhibiting positivity to the auto-antibodies in light of the frequent association of SCH with AITDs.

We observed a significant decrease in the concentration of TSH after 6 months of myo-Ins plus Se administration. Interestingly, the treatment restored physiological levels of TSH already after 3 months in a significant manner (**Figure 1**). While no relevant variation was observed in the values of the TH.

Additionally, the AIT index significantly improved after 3 months treatment and this trend is maintained over the 6 months of treatment, although without reaching significance (**Figure 2B**). The administration of myo-Ins and Se seems to effectively ameliorate the thyroid status by improving the AIT changes and restoring the value of TSH in the recognized normal range. Interestingly, several studies demonstrated that myo-Ins altered levels can affect inositol dependent TSH pathway, translating into impaired thyroid functionality, as observed in patients with hypothyroidism. The results of our study confirm that myo-Ins has a valuable activity in restoring the correct TSH signaling, impaired in SCH, maintaining a euthyroid status (1, 43, 44). Moreover, a significant decrease of the TSH levels over the treatment period, is still observed when the participants are stratified in two sub-groups: TSH value from 2.5 to 4.99 μIU/ml (group1), and TSH value from 5 - 10 μIU/ml (group 2). The treatment induced a significant reduction at T6 in group 1 (**Figure 1A**) and a significant decrease both at T3 and at T6 compared to baseline in group 2 (**Figure 1B**). This kind of stratification based on the TSH values represents an innovative aspect for the clinical studies focusing on the effect of the myo-Ins plus Se treatment in SCH patients.

Beside the AIT index, we also measured the thyroid volume over the treatment period as a benchmark of the thyroid inflammatory status. We observed that the thyroid volume remained unvaried after 6 months of treatment. However, a slight increase after the first 3 months was observed. One possible explanation is that Se and myo-Ins are unable to immediately reverse the effects of a pre-existing inflammatory state. However, in a six-month period of treatment the anti-oxidant effects of myo-Ins and Se seem to revert the mean (o median) thyroid volume to that observed at baseline.

In cases of hypothyroidism, a correlation with typical markers of autoimmunity is not uncommon (45). A subgroup of patients enrolled for the study exhibited positivity to the autoantibody TPO-Ab and Tg-Ab. The association of myo-Ins and Se significantly reduced the titer of both antibody titer measured over the treatment period. These data strengthen the evidence on the beneficial activity of myo-Ins and Se when administered in combination on the autoantibody levels (46). Indeed, increasing evidence describes a positive association of Se status and TH metabolism, and lower Se levels were observed in SCH patients compared to healthy subjects (47). To date, there are strong indications endorsing the effectiveness of Se supplementation especially in AITDs as Hashimoto thyroiditis, in reducing the antibody titer probably by influencing the immune system response and favoring the homeostasis of thyroid function (48, 49). AITDs are commonly characterized by a pro-inflammatory microenvironment mainly caused by the lymphocytic infiltration of the B and T cells, and by the accumulating levels of pro-inflammatory cytokines secreted (50). In these conditions, Se exhibits an important antioxidant activity which translates in radical scavenging properties and a valuable contribution for the reduction of the oxidative damages (51). Additionally, myo-Ins exhibits an important immune-modulatory effect that might be helpful in counteracting the lymphoid infiltration of the thyroid and in the regulation of the inflammatory state typically associated with autoimmune thyroiditis (52).

Over the treatment period, we observed a significant regularization of the menstrual cycle. Many authors described the importance of myo-Ins in the ovarian physiology, particularly in women with polycystic ovary syndrome (PCOS), which often exhibit irregular menstrual cycle due to alterations in the oogenesis and oocyte maturation, other than a hormonal unbalance (53, 54). Considering that thyroid dysfunctions may lead to menstrual cycle disturbances (38), we proved that myo-Ins supplementation is beneficial in ameliorating menstrual cycle regularity in women with hypothyroidism (**Figure 2A**). This improvement observed in menstrual cycle regularity, may be due to the activity of myo-Ins in the ovary, which amplifies the FSH signaling and favors the selection of the dominant follicle during the oogenesis process, effectively contributing to oocyte development (55, 56). Of note, there was no distinction for PCOS or not PCOS women enrolled in the study.

The observations made in the PCOS context has paved the way for the discovery of the beneficial effects of myo-Ins administration in patients affected by various forms of dysmetabolism (43, 57). In this regard, several studies investigated the efficacy of myo-Ins on lipid profiles among population with metabolic diseases demonstrating that supplementation helps decreasing triglycerides concentrations, as well as total- and low−density lipoprotein (LDL)–cholesterol levels (58). The same observations were confirmed in women affected by PCOS (59, 60), indicating that myo-Ins is an effective insulin sensitizer and strengthening the idea that its administration provides a useful approach for some metabolic disorders (44). The TH activity is able to influence the lipid levels through a genetic control or *via* increasing catabolism of LDL. High TSH values also correlates with hypercholesterolemia independently, and SCH condition seems frequently observed in hypercholesterolemic patients (61). The evaluation of total cholesterol concentrations carried out in the present study, as well revealed a significant improvement of its levels after 6 months of administration of myo-Ins and Se, thus indicating an efficacy of the treatment in the balancing of the lipidic species.

As thyroid altered network might affect the functions of different organs, a condition of hypothyroidism is commonly a biochemical definition, given the variability of clinical manifestation and of the related symptomatology (62). It should also be considered that in the earlier stages of thyroid disease, symptoms may remain unnoticed for prolonged periods (63). Nevertheless, a large percentage of patients with SCH already exhibit canonical signs and symptoms of hypothyroidism such as lethargy, weakness, cold intolerance, weight gain, fatigue, dry skin, and memory impairment (64). We provided participants a questionnaire to monitor the effects of the treatment on their quality of life and on the symptomatology related to the SCH pathology. The results revealed a positive response of the study group to the treatment with myo-Ins and Se with an overall improvement of the quality of life of the patients included in the study (**Figure 3**).

Data gathered from our study also confirm the safety profile of the supplementation with myo-Ins and Se. Noticeably, among all the patients enrolled for the study only a minor side effect was reported as a slight loss of appetite. Furthermore, all the individuals of the study group were highly compliant to the treatment. Taken together our results indicate an overall improvement of the SCH status for the patients enrolled in the study, and as well a significant decrease of discomfort related to the symptoms experienced with this pathological condition. A natural approach with dietary supplementation with myo-Ins plus Se seems able to counteract the manifestations of SCH and should be considered as a valid option to treat SCH with and without auto-immune features. Further study on a larger number of patients are required to confirm our findings and considering the absence of a control group in our experimental setting.

## Conclusion

The treatment of SCH is still debated giving the lack of conclusive evidence supporting the use of LT4 and the related issues of over prescription. On the other hand, the supplementation with myo-Ins and Se seems to effectively contribute to preserve euthyroid status, thus balancing the fluctuation of TSH, restoring the values of the physiological range, and ameliorating the quality of life of the patients. A huge number of data support the positive role of these two natural molecules in counteracting the typical features of SCH and of AITDs sometimes correlated to this condition. Even if numerous authors and researcher retrieved similar results regarding the administration of myo-Ins and Se, further evidence are still necessary to confirm these findings and to identify an approach commonly accepted to treat SCH.

## Data availability statement

The raw data supporting the conclusions of this article will be made available by the authors, without undue reservation.

## Ethics statement

The studies involving human participants were reviewed and approved by Ethical Comitee, National Institute of Endocrinology and Diabetology, Lubochna, Slovakia. The patients/participants provided their written informed consent to participate in this study.

## Author contributions

JP, PJ, MK, MZ and PV conceived the paper. All authors contributed to the article and approved the submitted version.

## Acknowledgments

The authors are extremely grateful to Dr. Orietta Picconi and Dr. Michele Russo for the helpful contribution with the statistical analysis and with the draft of the manuscript.

## Conflict of interest

MD is employee of Exeltis.

The remaining authors declare that the research was conducted in the absence of any commercial or financial relationships that could be constructed as a potential conflict of interest.

## Publisher’s note

All claims expressed in this article are solely those of the authors and do not necessarily represent those of their affiliated organizations, or those of the publisher, the editors and the reviewers. Any product that may be evaluated in this article, or claim that may be made by its manufacturer, is not guaranteed or endorsed by the publisher.
