# Supplementary material for: Supplementation with myo-inositol and Selenium improves the clinical conditions and biochemical features of women with or at risk for subclinical hypothyroidism
Source: Front Endocrinol (Lausanne). 2022 Nov 16;13:1067029. doi: 10.3389/fendo.2022.1067029 (PMC9709133; doi:10.3389/fendo.2022.1067029)
Supplement: Supplementary file 1 [file DataSheet_1.pdf]

Questionare of the lifestyle according to the following parameters

M0 ☐ /M3 ☐ /M6 ☐

M0 – preliminary visit M3 – control visit after 3 months M6 – control visit after 6 months

Initials o patient: __________ Year of birth: __________

Questions about quality of life

1. Evaluate your present status on scale 1 – 10 (1 = perfect, 10 = poor (harassing))

1	2	3	4	5	6	7	8	9	10

2. Evaluate level of fatigue of the scale (1 = perfect, 10 = poor (harassing))

1	2	3	4	5	6	7	8	9	10

3. Evaluate your tend to increase in weight or weight loss on the scale 1 – 10 (1 = perfect, 10 = poor (harassing))

1	2	3	4	5	6	7	8	9	10

4. Evaluate your cold or warm toleration on the scale 1 – 10 (1 = perfect, 10 = poor (harassing))

1	2	3	4	5	6	7	8	9	10

5. Evaluate your memory on the scale (1 = perfect, 10 = poor (harassing))

1	2	3	4	5	6	7	8	9	10

6. Evaluate your sweating on the scale (1 = perfect, 10 = poor (harassing))

1	2	3	4	5	6	7	8	9	10

7. Evaluate your swelling (edema) of soft tissue on the scale (1 = perfect, 10 = poor (harassing))

1	2	3	4	5	6	7	8	9	10

8. Evaluate status of your skin on the scale (1 = perfect, 10 = poor (harassing)))

1	2	3	4	5	6	7	8	9	10

9. Evaluate your "pins and needless" on the scale (1 = perfect, 10 = poor (harassing))

1	2	3	4	5	6	7	8	9	10

10. Evaluate your tolerance of loading on the scale (1 = perfect, 10 = poor (harassing))

1	2	3	4	5	6	7	8	9	10
